# Retraction: Spontaneous Rupture of Large Angiomyolipoma of the Kidney: A Rare Case

**DOI:** 10.7759/cureus.r102

**Published:** 2024-01-25

**Authors:** Rakan M Altuwayr, Fahad S Almutairi, Shuruq H Alkhaibari, Abdullah M Alharbi, Abdullah A Alramih, Raghdah A Alamri, Alwaleed S Alghamdi, Mishari M Alshammari, Thurya S Alshammari, Abdulmajeed A Alzahrani, Dina M Al Khafaji, Abdulrahman A Alamri, Khalid A Alshahrani, Sara A Binsalman, Faisal Al-Hawaj

**Affiliations:** 1 College of Medicine, Vision Colleges, Riyadh, SAU; 2 College of Medicine, Taif University, Taif, SAU; 3 College of Medicine, Qassim University, Buraydah, SAU; 4 College of Medicine, Dar Al Uloom University, Riyadh, SAU; 5 College of Medicine, Imam Abdulrahman Bin Faisal University, Dammam, SAU; 6 College of Medicine, King Abdulaziz University, Jeddah, SAU; 7 College of Medicine, Majmaah University, Al Majma'ah, SAU; 8 College of Medicine, University of Hail, Hail, SAU; 9 College of Medicine, Northern Border University, Arar, SAU; 10 College of Medicine, Dammam University, Dammam, SAU; 11 College of Medicine, King Khalid University, Abha, SAU; 12 College of Medicine, Ibn Sina National College For Medical Studies, Jeddah, SAU

The Editors-in-Chief have retracted this article. Concerns were raised regarding the identity of the authors on this article. Specifically, Faisal Alhaway and Malak Shammari have stated that they were added to this article without their knowledge or approval. The identity of the other authors could also not be verified. As the appropriate authorship of this work cannot be established, the Editors-in-Chief no longer have confidence in the results and conclusions of this article.

